# Peroxiporins in Cancer

**DOI:** 10.3390/ijms20061371

**Published:** 2019-03-19

**Authors:** Cecilia Prata, Silvana Hrelia, Diana Fiorentini

**Affiliations:** 1 Department of Pharmacy and Biotechnology, Alma Mater Studiorum, University of Bologna, Via Irnerio 48, 40126 Bologna, Italy; cecilia.prata@unibo.it; 2 Department for Life Quality Studies, Alma Mater Studiorum, University of Bologna, Corso D’Augusto 237, 47921 Rimini, Italy; silvana.hrelia@unibo.it

**Keywords:** aquaporins, peroxiporins, hydrogen peroxide, redox signaling, cancer, phytochemicals

## Abstract

The transport of H_2_O_2_ across membranes by specific aquaporins (AQPs) has been considered the last milestone in the timeline of hydrogen peroxide discoveries in biochemistry. According to its concentration and localization, H_2_O_2_ can be dangerous or acts as a signaling molecule in various cellular processes as either a paracrine (intercellular) and/or an autocrine (intracellular) signal. In this review, we investigate and critically examine the available information on AQP isoforms able to facilitate H_2_O_2_ across biological membranes (“peroxiporins”), focusing in particular on their role in cancer. Moreover, the ability of natural compounds to modulate expression and/or activity of peroxiporins is schematically reported and discussed.

## 1. Introduction

Aquaporins (AQPs), firstly identified by the Nobel Prize winner P. Agree [1,2], are membrane channels widely distributed in human tissues with different localizations at cellular and subcellular levels [3,4]. The 13 members (AQP0-12), belonging to this family, can be divided into three groups, according to their permeability profile and sequence homology. The classical water-selective channels, also called *orthodox* AQPs, include AQP0, AQP1, AQP2, AQP4, AQP5, AQP6, and AQP8. *Aquaglyceroporins* (i.e., AQP3, AQP7, AQP9, and AQP10) are permeable to water and some small uncharged solutes, e.g., glycerol, urea and gas of physiological importance [5,6,7,8]. Recently, a third subfamily was named s*uperaquaporins*, since its members, AQP11 and AQP12, have low homology at their amino acid level with other AQPs. For this reason, they are also called *unortodoxaquaporins* [9,10].

However, recent evidence shows that subfamilies functionally overlap, for example some AQP isoforms belonging to both orthodox and aquaglyceroporin are able to conduct ammonia [11,12].

AQPs are tetrameric proteins constituted by approximately 30 kDa monomers, each of which functions as an independent water channel. The monomer has six transmembrane helices, connected by five loops (A–E) and hydrophilic terminal amino and carboxyl groups are always located in the cytoplasmic side of the membrane. Moreover, the asparagine-proline-alanine (NPA) sequence is highly conserved [11,13]. Another conserved AQP structural feature is the aromatic/arginine (ar/R) constriction site located at the extracellular side of the channel. Having a diameter of 3 Å, which is only slightly larger than the 2.8 Å diameter of the water molecule, the ar/R constriction site is also called the “selectivity filter”. Aquaglyceroporins present a bigger pore size than water-selective aquaporins, since their diameter can reach the 3.4 Å dimension [11,14].

Recently, experimental evidence highlights that some AQPs allow also the transport of hydrogen peroxide (H_2_O_2_) through biological membranes; therefore, they are also named peroxiporins, as firstly suggested by Henzler and Steudle [15]. The expression, modulation and biophysical properties of molecular transport of AQPs are matters of intense investigation in all human organs and tissues. Moreover, a growing body of evidence indicates the involvement of AQPs in important pathophysiological functions, opening new diagnostic and therapeutic opportunities [14].

In particular, this review gives an overview of the emerging evidence on the functional involvement of peroxiporins in cancer progression, proliferation and metastasis.

Moreover, since the inverse relationship between the intake of different classes of dietary phytochemicals/nutraceuticals and the risk of cancer is established [16,17], a brief overview on the few experimental data about their modulatory effect on peroxiporins in cancer models is presented.

## 2. Peroxiporins

H_2_O_2_ belongs to the reactive oxygen species (ROS), known as oxidants that can react with various cellular targets thereby causing cell damage or even cell death. On the other hand, several works have demonstrated that H_2_O_2_ also functions as a signaling molecule controlling different essential processes in plants and mammals [18,19,20]. Because of these opposing functions, the cellular level of H_2_O_2_ is likely to be subjected to tight regulation *via* processes involved in its production, distribution and removal. Substantial progress has been made exploring the formation and scavenging of H_2_O_2_, whereas much remains to be learned about how this signal molecule is transported from its site of origin to the place of action or detoxification. 

Some members of the AQP family have been identified as candidates for the transport of H_2_O_2_ across membranes of mammalian cells and evidence is emerging about the physiological significance of aquaporin-facilitated H_2_O_2_ diffusion [21,22,23,24].

H_2_O_2_ is indeed well suited for redox signaling due to its relatively slow reactivity with biomolecules (second-order rate constants approximately 1 M^−1^·s^−1^). Nevertheless, there are some important exceptions, for example, oxidation of certain cysteinyl residues in some redox enzymes can proceed with high rate constant up to 10^8^ M^−1^·s^−1^ [25]. Therefore, these ox/red Cys residues can be considered as redox switches [26,27,28,29]. This high reactivity with specific Cys residues provides the basis on which H_2_O_2_ is engaged in intracellular signaling cascade. A spatiotemporal control of H_2_O_2_ levels could be obtained by different mechanisms such as activation/inactivation of H_2_O_2_ sources, the presence or absence of an adequate antioxidant system and, last but not least, the modulation of H_2_O_2_ transport across cellular membranes. It has been suggested that, when present at a physiological range of concentration (approximately 10 nM) [20,26], well below toxic levels, H_2_O_2_ is being utilized as a suitable second messenger in redox biology [27]. In this context, the important role that could be played by aquaporins is clear, in that they are able to control the passage of H_2_O_2_ through membrane, and in turn its cellular compartmentalization.

Recently, Almasalmeh and colleagues suggested that all water-permeable AQPs are H_2_O_2_ channels, although H_2_O_2_ permeability varies with the isoform with increasing H_2_O_2_ permeability from aquaglyceroporins *via* orthodox AQPs to aquaammoniaporins. The latter displays the optimal pore layout for H_2_O_2_ transport, as a result of intermediate diameters in conjunction with high water permeability [28]. To date, only some of them have been shown to be permeable to H_2_O_2_ [22,23,29,30,31,32]: the aquaporin/aquaammoniaporin AQP8, the aquaglyceroporins AQP3 and AQP9 but probably also AQP11, frequently defined as unorthodox aquaporin, due to its distinct evolutionary pathway [9]. AQP5 as well is supposed to be a peroxiporin with a crucial role in tumorigenesis [33].

The permeability coefficient of H_2_O_2_ was found to be very close to that of water, and treatment with HgCl_2_ significantly decreased the influx of both molecules into the cells, as well as silver ions that abolished the aquaporin-mediated H_2_O_2_ sensitivity of yeast cells [22,23]. The structural requirements for H_2_O_2_ passage through AQP proteins are not yet completely established, although emerging studies have been performed in order to elucidate this aspect regarding the different isoform of AQPs/peroxiporins.

Different AQPs with a different localization and different function could be present in the same cell type. For example, AQP3, -7, -8, and -11 proteins were found in human sperm cells and localized in the head (AQP7), in the middle piece (AQP8) and in the tail (AQP3 and -11) in both the plasma membrane and in intracellular structures. Their function has been related to both volume regulation and ROS elimination [4]. 

Besides the involvement of peroxiporins in ROS elimination from cell compartments, recent but numerous evidence suggests their essential role in channeling Nox-derived H_2_O_2_, as previously demonstrated in a model of human acute myeloid leukemia [31] cell line and also in other different experimental models [34].

## 3. Redox Signaling in Cancer

Redox signaling has fundamental implications in the pathophysiology of cells and organisms in a similar way to other well-characterized signaling pathways (e.g., those mediated by hormones, calcium, etc.) and, in particular, Moloney and Cotter recently wrote a very comprehensive review about the role exerted by ROS in cancer biology [35]. 

The key second messenger in redox regulation is hydrogen peroxide. Specialized protein cysteines can undergo many redox-dependent modifications providing versatile switches for tuning protein activity/inactivity and hence influencing a wide variety of cellular pathways linked to proliferation, differentiation, tissue repair, inflammation, circadian rhythm, and aging [20]. Redox signaling encompasses both the physiological and pathological roles of ROS. As well described and highlighted by Sies and Colleagues, ROS (in particular H_2_O_2_), could be involved either in reversible “oxidative eustress” linked to redox regulation of physiological processes or in the damage to biomolecules (“oxidative distress”) Besides the known detrimental role, hydrogen peroxide indeed emerged as major redox metabolite operative in redox signaling and redox regulation, according to its concentration, compartmentalization and spatio-temporal localization. Under physiological conditions (1–10 nM), H_2_O_2_ is involved in the homeostatic redox signaling, denoted as oxidative eustress. Higher concentrations lead to adaptive stress responses by the involvement of Nrf2/Keap1 or NF-κB. Supraphysiological concentrations of H_2_O_2_ (>100 nM) lead to damage of biomolecules, denoted as oxidative distress [36].

It is well known that cancer cells are frequently characterized by increased levels of ROS related to a constitutive activation of growth factor pathways to sustain cellular growth and proliferation, in comparison to their normal counterparts [37]. The high ROS at intracellular level, detected in various cancer types, has been shown to have several roles, for example, they can activate pro-tumourigenic signaling, enhance cell survival and proliferation, and drive DNA damage and genetic instability [35]. Growth factors activate the intrinsic tyrosine kinase activity of their receptors (RTKs), which in turn activate several key signal transduction pathways, such as PI3K-Akt signaling and RAS-MEK-ERK-MAP kinase cascade, to promote cell proliferation, nutrient uptake and cell survival [38,39,40,41,42,43,44,45,46,47,48]. Protein kinases (e.g., RTKs and PI3K) are negatively regulated by protein tyrosine phosphatases (PTPs), resulting in the dampening of mitogenic signaling. 

Catalytic cysteine of protein tyrosine phosphatases can, in turn, be inactivated by H_2_O_2_ derived by Nox induced by growth factors [48,49]. Noxs have been confirmed to be closely correlated with cancer development, progression, proliferation and metastasis through positive feedback redox regulation of PI3K/Akt signaling. ROS are also generated from increased oxidative metabolism and hypoxia in rapidly expanding tumors. Besides, cancer cells express elevated levels of cellular antioxidants (SODs, GSH, GPx, PRx) in part mediated by nuclear factor erythroid 2-related factor 2 (Nrf-2) to protect against oxidative stress-induced cell death [47]. 

Aside from the specificity and selectivity of ROS on their targets, the compartmentalization of ROS production within cells is an important determinant. Effective redox signaling requires that H_2_O_2_-dependent oxidation of a given protein is likely to occur close to the source of H_2_O_2_ production. For example, the protein targets of H_2_O_2_ generated from plasma membrane Nox are also located at/nearby the plasma membrane [47]. Mitochondria, one of the main contributors to the endogenous ROS generation, have up to 10 known sites capable of generating superoxide. However, the relative contribution of each of these sites to the production of superoxide remains largely unknown [35]. Most of the superoxide generated inside the mitochondria is dismutated to H_2_O_2_ by manganese superoxide dismutase (MnSOD) in the mitochondrial matrix [50]. H_2_O_2_ has been shown to cross the mitochondrial membrane through specific members of the aquaporin family [23]. Deregulation of mitochondrial ROS can result in the initiation and progression of various cancer types. Some cancers, associated with elevated mitochondrial ROS levels, include chronic lymphocytic leukemia [51], acute myeloid leukemia [52] and breast cancer [53]. Moreover, mitochondria, are known to move dynamically towards their targets, thus allowing mitochondrially generated H_2_O_2_ to activate specific signaling pathways [54].

In summary, determining whether an anti-cancer therapy should focus on lowering ROS levels to prevent cell proliferation or on strong increasing ROS to kill cancer cells remains the central question in redox biology. However, if the ROS levels are not sufficiently raised within tumor cells, therapy would simply further activate NF-kB, PI3K, HIFs, and MAP kinases to promote tumorigenesis; moreover, agents used to disabling antioxidants should act only in cancer cells without disabling antioxidants in normal cells [55,56,57]. Interestingly, since different cancer cell types have varying peroxiporin expressions, variation in peroxiporin expression could alter cell susceptibility to therapeutic H_2_O_2_ concentrations [58].

In this context, AQPs represent new and promising anticancer targets, since the channel-mediated membrane transport permits the fine adjustment of H_2_O_2_ levels in cell compartments, allowing it to function as a signal molecule [22].

Several studies indeed reported the expression of AQPs in a variety of human tumors, which in some cases are correlated with tumor grade [59].

## 4. Peroxiporin 3 and Cancer

AQP3 is permeable to water, glycerol and urea but also to H_2_O_2_, as recently demonstrated [22,28,60]. AQP3 is widely distributed in human tissues, representing the most abundant skin aquaglyceroporins and one of the isoforms with an important role in kidney [11]. 

The role of AQP3 as peroxiporin was recently reported in different papers regarding the role played by H_2_O_2_ transport across the plasma membrane. T cell migration toward chemokines resulted in being dependent on AQP3-mediated hydrogen peroxide uptake [61] and AQP3 was shown to mediate H_2_O_2_ transport correlated to inflammation in keratinocytes and the development of psoriasis [62]. Moreover, the AQP3-mediated transport of H_2_O_2_ through the plasma membrane likely plays a pivotal role in cancer progression [30,63].

Many reports suggest the existence of a possible relationship between AQP3 expression and cancer progression or prognosis in many neoplastic tissues [64]. Overexpression of AQP3 and AQP5 were found in hepatocellular carcinoma [65], esophageal squamous cell carcinoma [66] and pancreatic ductal adenocarcinoma [67], compared with paired adjacent non-neoplastic tissues. It was also observed that the increased expression of both AQP isoforms was associated with tumor stage, grade, metastasis and patient prognosis, concluding that this AQP3-AQP5 co-expression could be a useful diagnostic and prognostic marker for these types of malignancy. Recently, the overexpression of AQP3-AQP5 has been considered as a therapeutic marker in patients with triple-negative breast cancer [68]. A much higher AQP3 expression than in normal tissues occurred also in colorectal carcinoma [69] and AQP3 overexpression was reported in prostate cancer cells [70]. In these cells, AQP3 may modulate motility and invasion *via* regulation of ERK1/2-mediated matrix metalloproteinase-3 secretion, as previously observed in human gastric cancer cells [71]. 

The expression of AQP3 is stimulated by hormones that are commonly increased in cancer, such as EGF and estrogens, but how overexpressed AQP3 accelerates cancer progression is not clear. The role of AQP3 in EGFR-dependent cell signaling and cancer progression was investigated in human squamous cell carcinoma and human lung cancer cell lines, both expressing high concentrations of AQP3 and EGFR [30]. The results show that AQP3 is required for EGF-induced cell signaling and cancer progression by a mechanism involving EGF-induced generation of extracellular H_2_O_2_, and its intracellular transport by AQP3. As previously reported, on the plasma membrane of mouse primary keratinocytes [62] a co-localization of AQP3 with EGFR and Nox2 was observed in squamous cells [30]. This finding indicates that AQP3 and EGFR form a signaling module that triggers Nox activation to generate the second messenger H_2_O_2_ which is transported into cells by an AQP3-mediated channeling. An association between Nox2 and another peroxiporin, AQP8, was reported also by Prata and Colleagues, who described the existence of VEGF-activated Nox2-AQP8 axis in a leukemic cell line [72]. The evidence of the interaction between AQP isoforms and Nox2 supports the importance of these two partners in the redox signaling cascade. Moreover, Hara-Chikuma and Colleagues [30] detailed also that the protein tyrosine phosphatases PTEN and SHP2 are downstream targets for AQP3-mediated cellular H_2_O_2_ within EGF-induced cell signaling. These findings indicate AQP3 as a potential new therapeutic target in cancer therapy involving EGF/EGFR cell signaling.

The involvement of AQP3 was also demonstrated in human breast cancer cell lines expressing CXCR4 but not EGFR [63]. Binding of the chemokine CXCL12 to its receptor CXCR4 stimulates downstream G protein signaling, leading to the activation of the PI3K/Akt or MAPK pathway, and the CXCL12/CXCR4 axis is a key step in breast cancer cell migration toward the lungs [73]. In these cells, H_2_O_2_ was produced in the extracellular space *via* Nox2 in response to CXCL12 stimulation, then rapidly transported into breast cancer cells through AQP3. Also in this case, a complex between AQP3 and Nox2 was found at the leading edge in polarized breast cancer cells, where a transient H_2_O_2_ accumulation occurred during CXCL12-induced chemotaxis. Thus, also in breast cancer cells, the Nox2/AQP3 complex is required for H_2_O_2_ intracellular transport and for H_2_O_2_ to exert its downstream cell-signaling effects. Downstream effects exerted by Nox2-induced AQP3-transported H_2_O_2_ were both PTEN/PTP1B oxidation and Akt phosphorylation in response to CXCL12 stimulation, directly activating the PI3K/Akt pathway and leading to cell survival, proliferation and migration. These data suggest the inhibition of AQP3 expression or activity as a promising tool in limiting cancer cell metastasis [63].

## 5. Peroxiporin 5 and Cancer

Besides its known expression in salivary and lacrimal glands, AQP5 was found to be overexpressed in cancer cells and tumor tissues, suggesting that it may be implicated in tumor formation, cell proliferation, migration and survival through multiple pathways that are not yet fully understood [33]. In particular, it has been suggested that AQP5 involvement in intracellular signaling transduction pathways are implicated in tumor formation [74]. Therefore, the mechanism explaining AQP5 contribution to tumorigenesis could be its ability to facilitate H_2_O_2_ channeling through the plasma membrane, as suggested by Direito and colleagues [33]. The contrasting phosphorylation status observed in normal tissues and cancer cells, where AQP5 was found preferentially in the phosphorylated form on Ser156 and Thr259, suggests that AQP5 role in tumorigenesis is related also to its phosphorylation [33,75,76]. 

It is also known that the AQP5 channel can switch between different conformations characterized by distinct rates of water flux. The transition between open and closed states involves the displacements of Hys67 residue and the orientation of His173 residue, located near the ar/R selectivity filer, allows the transition between wide and narrow states. Nevertheless, the trigger for AQP5 gating still remains unclear [77]. 

In the yeast-expressing rat AQP5, Rodrigues and Colleagues [20] demonstrated that AQP5 is able to mediate H_2_O_2_ diffusion through plasma membranes, influencing signaling transduction pathways involved in tumorigenesis. In addition, their data show that PKA-dependent phosphorylation, simultaneously stimulates AQP5 trafficking and abundance at the plasma membrane and contributes to the opening of its channel at physiological pH 7.4. 

## 6. Peroxiporin 8 and Cancer

Several studies demonstrated that AQP8 transports water [8,78,79,80,81] and ammonia [8,82] but it is also able to transport H_2_O_2_ across membranes [4,22,23,28,29,31,34,83,84]. AQP8 is predominantly distributed in gastrointestinal epithelial cells, liver, pancreas, and male/female reproductive systems [85]. Evidence has been reported about AQP8 involvement in human esophageal [85] and cervical cancer [86], both proceeding *via* the EGFR/ERK1/2 pathway. 

Furthermore, a role for AQP8 has been described in many human cancer cell lines. In HeLa cells, a line derived from a human cervical cancer, AQP8 allows entry of EGF-induced H_2_O_2_ exogenously produced, thus amplifying signal transduction and affecting downstream tyrosine phosphorylation [28]. These data indicate that H_2_O_2_ transporters may be potential targets in cancer diseases.

Evidence has been provided about the important role of AQP8 in H_2_O_2_-mediated redox signaling linked to leukaemia cell proliferation and survival [31,84]. In a cell line derived from a human acute myelogenous leukemia, endogenous VEGF triggers Nox-derived H_2_O_2_ production, which provokes VEGFR-2 phosphorylation and the consequent modulation of many cellular activities, resulting in cell survival and proliferation [38]. By means of a dimedone-based method for easily monitoring cellular protein sulfenation, our group demonstrated, for the first time, the role of AQP8 on the fine tuning of cysteine oxidation in target proteins involved in leukaemia cell proliferation pathways [84]. In particular, when cells were exposed to exogenous H_2_O_2_, AQP8 overexpression enables the formation of high intracellular level of oxidized cysteines, whereas AQP8 silencing diminished the downstream VEGF signaling. In AQP8-silenced cells, the downstream targets, such as the protein tyrosine phosphatases PTEN and Akt, were found mainly in the cysteine-reduced and non phosphorylated form, respectively. These findings suggest that the development of new drugs targeting specific AQP isoforms might be an interesting novel antileukemia strategy. 

The role of AQP8 in assuring efficient cytosolic import of H_2_O_2_ likely produced by Noxs has been well documented, and therefore many investigations aiming to a deeper insight into the underlying mechanism have been carried out. It has been demonstrated that AQP8-dependent H_2_O_2_ transport in HeLa cells can be fine-tuned during stress conditions known to involve ROS production [83]. These results show that different stresses converge in gating AQP8-mediated H_2_O_2_ transport *via* reversible oxidation on cysteine C53, a highly conserved residue in all the AQP isoforms able to transport H_2_O_2_ [83]. In a following paper, AQP8 gating by persulfidation of cysteine C53 has been shown [87]. Upon H_2_O_2_ entry into AQP8 channel, cysteine C53 is oxidized, being thus primed for persulfidation by H_2_S locally produced by cystathionine-β-synthase (CBS). The additional sulfur atom modifies the narrow conducting channel, preventing H_2_O_2_ transport. The presence of the highly conserved cys C53 in peroxiporins suggests that a similar mechanism of gating could be operating in all the H_2_O_2_-transporting AQPs, and persulfidation of highly reactive cysteins might result in channel closure [87]. Through this mechanism cells can integrate different signaling pathways in order to efficiently adapt to environmental changes. 

AQP8 was also found to be localized at inner mitochondrial membrane in the liver and initially hypothesised to be involved in water transport [78]; nevertheless, a later study did not find differences in water permeability between wild-type and AQP8-deleted mice hepatic inner mitochondrial membranes [88]. Interestingly, other Autors suggested a potential important role in H_2_O_2_ channelling across the mitochondrial membrane: Marchissio and Coworkers showed that mitochondrial aquaporin-8 knockdown in human hepatoma HepG2 cells causes ROS-induced mitochondrial depolarization and loss of viability [24,89] and Danielli and Coworkers showed that hepatocytic mitochondrial AQP8 works as a multifunctional membrane channel protein that facilitates the uptake of ammonia for its detoxification to urea as well as the mitochondrial release of hydrogen peroxide. Moreover, their results suggested that cholesterol can regulate transcriptionally human hepatocyte mitochondrial AQP8 expression, likely *via* SREBPs [90].

## 7. Peroxiporin 9 and Cancer

AQP9 is expressed in many cell types including hepatocytes, where it is involved in the uptake of NH_3_ and in the efflux of newly synthesized urea [91]. AQP9 is also permeable to water, glycerol, carbamides, CO_2_, antimonite, arsenite, and other larger molecules such as lactate, pyruvate, purine, pyrimidine [11,92], probably due to a larger pore observed by 3D structure analyses [93]. However, much remains to be learned about AQP9 modulation and function. A recent report shows that AQP9 facilitates membrane transport of hydrogen peroxide in mammalian cells [32]. These authors showed also that deficiency of AQP9 decreased H_2_O_2_-induced cytotoxicity, suggesting an involvement of AQP9 in redox signaling. The potential phosphorylation sites located at Ser28, Ser11 and Ser222, probably representing PKC targets, have been suggested for the human isoform [94,95], supporting the hypothesis of AQP9 involvement in the redox signaling processes.

## 8. Peroxiporin 11 and Cancer

The ”unorthodox” or “superaquaporin” AQP11 is mainly localized at the endoplasmic reticulum (ER) and is permeable to water as well as glycerol [10]. It plays an important role in preventing glucose-induced oxidative stress in kidney proximal tubules but it has not been yet directly demonstrated to facilitate the diffusion of H_2_O_2_ through membranes [96]. AQP11 presents a unique aminoacidic sequence pattern (Asn-Pro-Cys) which appears essential for the molecular function [97]. Very recently, Hoshino Y. and Colleagues suggested that Nox2-induced oxidative stress caused by the reduced function of AQP11 might be relevant to the pathology of diabetic nephropathy and renal allograft dysfunction [98]. It is plausible that AQP11 was controlling intracellular ROS accumulation by acting as an endoplasmic reticulum H_2_O_2_ channel [99].

Taken together, these highlights suggest that peroxiporins have gained an important role as fine level regulators in the transduction of redox signals and the possibility of their regulation seems of great interest. Molecules able to modulate the activity and/or expression of peroxiporin isoforms could be useful tools to influence the cellular response and could represent unforeseen therapeutic opportunities.

## 9. Development of Novel Therapeutic Strategies by Means of Natural Compounds Able to Modulate Peroxiporin Expression/Activity in Cancer

Several molecules present in food and edible plants (phytochemicals recognized as safe by the American Food and Drug Administration and European Food Safety Agency) have been found to prevent, delay and/or co-treat many human diseases, by modulating important cell functions. Beside the great impact on health status, this is an important cost-effective opportunity for decreasing the large burden of health care, associated with chronic diseases [100].

The modulatory effects on AQPs exerted by food bioactive phytocompounds is a new and intriguing scientific field of interest [101,102] and some of these compounds seem to act at different molecular and cellular levels to modulate peroxiporins involved in cancer (Table 1). 

It has been demonstrated that EGF-induced cell migration plays important roles in ovarian cancer invasion and metastasis [106] and that curcumin, one of the most well-known natural compound having chemopreventive potential, has to be considered as a beneficial support in ovarian cancer treatment strategies [107]. Moreover, recent evidence has shown that curcumin is able to suppress EGFR signaling in prostate cancer [108]. Ji and Coworkers demonstrated that EGF, *via* the EGFR signal transduction pathway, induces AQP3 expression, which is involved in cell migration in CaOV3 cells [103]. Curcumin treatment alone down-regulates AQP3 expression in CaOV3 cells, furthermore, it was able to both inhibit EGF-induced AQP3 up-regulation and block EGFR downstream signaling, thus diminishing cell migration. It was concluded that the anti-cancer activities of curcumin might, at least in part, be due to its potent ability to down-regulate AQP3 content in human ovarian cancer cells and that EGF/EGFR, PI3K/Akt and MEK/ERK signaling pathways are involved in EGF-induced AQP3 expression and cell migration in CaOV3 cells. Although the authors do not suggest an involvement of H_2_O_2_ in the curcumin-inhibited EGF/EGFR signaling in CaOV3 cells, it is known that EGF signals by a mechanism involving EGF-induced generation of extracellular H_2_O_2_ and its intracellular transport by the peroxiporin AQP3, as demonstrated in [30]. 

The effect of five selected antioxidant compounds (ASME, Curcumin, Marrubiin, Naringenin and Quercetin) on the water/hydrogen peroxide permeability of HeLa cells (expressing AQP1, AQP3, AQP8 and AQP11) was studied in the presence or absence of oxidative stress conditions [104]. Indeed, water movement changes are considered representative of an H_2_O_2_ flux variation [4,83]. Owing to their antioxidant activity, these compounds are able to normalize the H_2_O_2_ permeability of HeLa cells when added during or after the stressor stimulus. As reported above, it has been demonstrated that AQP8-dependent H_2_O_2_ transport in HeLa cells can be fine-tuned during stress conditions *via* reversible oxidation on cysteine C53 [83]. Unexpectedly, curcumin did not protect nor restore the AQP’s permeability properties in stressed cells, but showed a direct inhibitory effect on AQP isoforms. This result appears of great interest, since few potential AQP inhibitors have been identified to date, some of them having high toxicity and low specificity.

Human ovarian cancer and ascite production is related to an increase of AQP5 expression, as demonstrated in SKOV3 and CaOV3 cells [109]. Epigallocatechin gallate (EGCG) has been reported to have anticancer activity, particularly inhibiting cell proliferation and apoptosis [110]. Yan and Coworkers observed that EGCG inhibited the growth of SKOV3 in a dose- and time-dependent manner and, upon this cell treatment, AQP5 expression was gradually decreased, indicating that AQP5 expression is related to SKOV3 cell growth [105]. Regarding the EGCG mechanism, they found that EGCG inhibited the nuclear expression of NF-κB, thus demonstrating that the block of NF-κB activation can inhibit the expression of AQP5 and the transcription of its gene, suggesting that NF-κB can regulate AQP5 expression in SKOV3 cells, in agreement to [111], reporting this regulation in mouse lung epithelial cells. 

Prata and colleagues investigated the potential effect of the isothiocyanate sulforaphane (SFN) on the modulation of AQP8 and Nox expression in a model of human acute leukemia [72], since it was stated that SFN is able to selectively exert cytotoxic effects in many cancer types or being even cytoprotective in normal cells [112]. The results show that SFN (at concentrations readily reachable through dietary intake, i.e., 10 μM) downregulates AQP8 and Nox2 expression, limiting both H_2_O_2_ production and entry into the cells. By using a co-immunoprecipitation technique, it was also demonstrated that Nox2 and AQP8 are linked to each other, thus, by decreasing the effect of this Nox2-AQP8 axis, SFN causes profound effects on the redox signaling, survival and proliferation of these leukemic cells [72].

The list of phytochemicals/nutraceutical compounds modulating AQPs is far from complete. Further efforts have to be made in order to identify new natural, chemopreventive agents and work is also needed to validate in humans the results that have been obtained in vitro or in animal models. In addressing the action of polyphenols or other natural compounds on AQP expression, it is also important to keep into consideration that, frequently, these compounds are not active per se since their metabolites are frequently the chemical entities endowed with bioactivity. 

Pharmacological ascorbate therapy [58] relies on the ability of peroxiporins to channel H_2_O_2_ through the plasma membrane. This therapy has been indicated as adjuvant in the treatment of pancreatic ductal adenocarcinoma [113]. It has been recognized that the primary responsible for ascorbate cytotoxicity is H_2_O_2_, formed extracellularly as a byproduct of ascorbate oxidation, and then permeating into the intracellular space through peroxiporin channeling [114]. Unlike normal cells, which are relatively unaffected by ascorbate, cancer cells exhibit a wide range of susceptibility, depending on their catalase activity and plasma membrane permeability to H_2_O_2_. Erudaitius and colleagues demonstrated that silencing of AQP3 in pancreatic cells prevented the accumulation of lethal intracellular H_2_O_2_ concentrations and resulted in an increased surviving fraction of pancreatic cells in a clonogenic assay [58]. This implies that cell-susceptibility to ascorbate therapy is significantly coupled to the plasma membrane permeability to H_2_O_2_, and, consequently, to elevated expression of peroxiporins, often a hallmark of cancer cells. 

The therapeutic strategy, in this case, relies in increasing the level of AQP3 expression, and it is interesting to observe that gemcitabine, a drug used for the control of metastatic and node-positive pancreatic cancer [115], has been demonstrated to stimulate AQP3 expression [116].

## 10. Conclusions

Efficient and regulated transmembrane diffusion of H_2_O_2_, a key molecule in the redox signaling network, requires aquaporins and makes these channels important players in this signaling process in both normal and transformed cells, as schematically reported in the Graphical Abstract. 

AQP expression in tumors has also been proposed to be of diagnostic and prognostic value [21,32], since AQPs are heavily expressed in many types of tumors [117], especially those considered aggressive [37]. 

Experimental evidence supports AQPs as possible “druggable” proteins [118]. Indeed, phytochemicals gained remarkable attention in cancer chemoprevention, due to their pleiotropic roles and mechanisms of action, but great consideration must be devoted to their bioavailability [119] and to the possible interference in drug pharmacokinetics/pharmacodynamics [120]. 

To exploit the potency of phytochemicals and nutraceuticals as AQP modulators in cancer, there is a need for new experiments designed specifically to completely clarify the involvement of AQPs in cancer progression and the identification of their role in hallmarks of cancer development.

## Figures and Tables

**Table 1:** Phytochemicals/nutraceutical compounds modulating aquaporins (AQPs) in cancer.

Natural Compounds	Experimental Model	AQP Isoforms	Anticancer Effect	Reference
Curcumin	CaOV3	AQP3 ↓	↓ cancer cell migration	[103]
Curcumin	HeLa cells	AQP1, AQP3, AQP8, AQP11 (ND)	↓ H_2_O_2_	[104]
ASME, Marrubiin, Naringenin, Quercetin	HeLa cells	AQP1, AQP3, AQP8, AQP11 (ND)	↓ H_2_O_2_	[104]
EGCG	SKOV3	↓ AQP5	↓ proliferation ↑ apoptosis	[105]
Sulforaphane	AML cells	↓ AQP8	↓ H_2_O_2_ ↓ proliferation	[72]

↓: decreased; ↑ increased.

## Abbreviations

AQP	aquaporin
ROS	reactive oxygen species
RTK	tyrosine kinase receptor
PTPs	protein tyrosine phosphatases
Nox	NAD(P)H oxidase family
SMC	smooth muscle cell
NF-kB	nuclear factor kappa-light-chain-enhancer of activated B cells
EGF/EGFR	epidermal growth factor and its receptor
VEGF/VEGFR	vascular endothelium growth factor and its receptor
ASME	(*R*)-aloesaponol III 8-methyl ether
EGCG	epigallocatechin gallate
SFN	sulforaphane
